# Who’s been framed? Framing effects are reduced in financial gambles made for others

**DOI:** 10.1186/s40359-015-0067-2

**Published:** 2015-04-02

**Authors:** Fenja V Ziegler, Richard J Tunney

**Affiliations:** School of Psychology, University of Lincoln, Brayford Pool, Lincoln, LN6 7TS United Kingdom; School of Psychology, University of Nottingham, Nottingham, NG7 2RD United Kingdom

## Abstract

**Background:**

Decisions made on behalf of other people are sometimes more rational than those made for oneself. In this study we used a monetary gambling task to ask if the framing effect in decision-making is reduced in surrogate decision-making.

**Methods:**

Participants made a series of choices between a predetermined sure option and a risky gambling option of winning a proportion of an initial stake. Trials were presented as either a gain or a loss relative to that initial stake. In half of the trials participants made choices to earn money for themselves and in the other half they earned money for another participant. Framing effects were measured as risk seeking in loss frames and risk aversion in gain frames.

**Results:**

Significant framing effects were observed both in trials in which participants earned money for themselves and those in which they earned money for another person; however, these framing effects were significantly reduced when making decisions for another person. It appears that the reduced emotional involvement when the decision-maker is not affected by the outcome of the decision thus lessens the framing effect without eradicating it altogether.

**Conclusions:**

This suggests that the deviation from rational choices in decision-making can be significantly reduced when the emotional impact on the decision maker is lessened. These results are discussed in relation to Somatic Distortion Theory.

## Background

Recent research suggests that framing effects may be the result of distortions in probability estimation that result from the interplay between emotional processing and decision-making (De Martino et al. 2006; Tunney & Ziegler, submitted). Framing effects occur when the decisions that people make change as a result of the way in which the outcomes are described to the participant. Typically, framing effects are revealed as aversions to risk when gambles are presented as gains, and preferences for risk when presented as losses. The classic example is the ‘Disease Problem’ (Tversky and Kahneman 1981) in which the participants were presented with a vignette describing the choices available to a government in preparing for a disease pandemic. In the gain frame the choice is between a ‘sure’ option of saving some, but not all lives, and a ‘risky’ option with a chance that everyone will be saved but a chance also that everyone will die. The expected utilities in both frames are identical but described slightly differently. In the loss frame the outcomes are worded differently; in the ‘sure’ option, some but not all lives will be lost; and in the risky option there is a chance that all lives will be lost, and a chance that none will be lost. The basic result is that participants tend to prefer the sure option of definitely saving some people in the gain frame. However, when presented with the loss frame, participants tend to prefer the risky choice. In this design a framing effect is revealed as a kind of preference reversal. This variance in decision-making for essentially the same problem is a clear and robust violation of rational choice theory (see Mellers et al. 1998). On reflection it should come as no surprise that emotion would play some part in decisions about vignettes that involve the hypothetical death of thousands of people, or more trivially about real financial rewards. Almost inevitably the choices that we make in these sorts of scenarios are likely to be affected by our emotional expectations of the outcomes (a simulated projection of how we would feel given the outcome), in addition to the analytic computation of the problem. This may be the result of an affect heuristic (Slovic et al. 2002) or a distortion in the estimate of subjective probability (Tunney & Ziegler submitted). It follows that if we are not emotionally involved in the outcomes of a decision then these framing effects should be reduced or disappear entirely. In this paper we report an experiment in which participants made a series of choices between a sure outcome and a gamble in both gain and loss frames. Our key manipulation was that on half the trials the participant was the financial beneficiary of the outcome of the decisions, while on the other half the beneficiary was another person.

Until recently research has almost exclusively focused on decisions people make for themselves. Whilst these are likely to form the majority of decisions that we make, there are nonetheless a sizeable number of decisions we make for someone else in our personal, social and professional lives. Would we expect to see a difference between decisions we make for ourselves compared to those we make for others? According to the risk-as-feeling hypothesis (Loewenstein et al. 2001) risk is processed as a feeling of anticipation or fear, rather than an objective numerical value. Experiencing this visceral or somatic responses to risk puts the decision-maker into a hot state of cognition, but because of interpersonal empathy gaps the emotional response of another person to risk is underestimated (Loewenstein 1996). As a consequence, it can be predicted that decision-makers would make decisions less influenced by the emotional response to risk for other people, in other words, decisions for self and others under risk would be different.

However, results from several studies investigating the difference between self and other risky decisions are far from clear-cut. Although differences is self-other decision making have been observed in non-financial risky decisions (Stone and Allgaier 2008; Stone et al. 2013). Stone et al. (2002) summarized the literature as generally not showing a difference in decisions involving monetary payoffs under risk which are made for self and other. This is perhaps counterintuitive, as the risk-as-feeling hypothesis would predict that with the reduced emotional impact of a decision where the outcome does not affect the decision maker, he or she becomes more risk seeking. And indeed that is the result from Hsee and Weber’s (1997) landmark study on the framing effect in self and other decisions. When predicting the decisions another person would make, participants were more risk seeking than in decisions made for themselves (Hsee and Weber 1997). However, Hadar and Fischer (2008) found the opposite pattern: participants were more risk seeking in decisions made for themselves than predictions for others, especially when risks were high and the decisions were reciprocal. And, in a third pattern, Benjamin and Robbins (2007) found no difference in risk seeking behaviour in self and other decisions in a task which involved hypothetical payoffs.

Why might we see such differences in decisions made for another person? Ziegler and Tunney (2012) predicted that the relative social or emotional distance to other person plays a pivotal role. They asked participants to make a series of decisions, choosing between a smaller immediate reward and a larger delayed reward. When the decision was made for themselves participants were more impulsive than if the decision was made for more socially distant people. Ziegler and Tunney modeled social distance based on Wright’s (1922) coefficient of relatedness and found that impulsivity declined systematically with the distance of the relationship and was lowest for a complete stranger, but very high for a best friend. The reduced impact of receiving an immediate reward appeared to improve participants’ ability of self-control, leading to decisions that were closer to a normative choice optimum. Tunney and Ziegler (submitted) used this result in conjunction with a careful analysis of the surrogate decision-making literature to formulate the Somatic Distortion theory of surrogate decision-making, which attempts to capture the potential conflicts between the different perspectives one might encounter when making a decision on behalf of another person, along with the familiarity between decision-maker and recipient, and the individual differences in the factors that might affect perspective taking. In doing so the model predicts when a decision made for another person is different from a decision made for ourselves. The model assumes that when making a decision on behalf of another person, the decision maker compares the preferred outcome from a number of different perspectives. The decision maker considers what they would do if they were the beneficiary of the decision, they simulate what they believe the beneficiary would choose, and what they believe what the best outcome for the beneficiary would be irrespective of their own wishes or the beneficiaries. We refer to each perspective as Projected, Simulated, and Benevolent respectively. Of course, the decision-maker might simply decide what they would prefer to outcome to be irrespective of either the beneficiaries wishes, or indeed what they would chose if they were the beneficiary. In this egocentric scenario the decision maker does not attempt to make a surrogate decision and the theory does not apply. In computing a decision from each perspective the decision maker makes an estimated utility judgment. The subjective utility estimation is distorted as a function of the participants’ knowledge or familiarity with the beneficiary. Given that what we would choose might be different from what we think another person might choose, and that both perspective could be different again from what we believe the best choice (the benevolent choice) to be, the model includes a simple majority choice rule to decide what the actual surrogate decision would be. Of course, perspective taking is an individual difference and we expect that the accuracy of a surrogate utility judgment will vary not only as a function of external factors such as familiarity, but also of internal factors such as ones ability to take another person’s perspective.

Instances in the literature when participants were predicting what another person *should* (e.g. Ziegler and Tunney 2012) or *would* (e.g. Hsee and Weber 1997) do is not the same as making actual decisions on behalf of another person. This study seeks to investigate, firstly, how people actually make decisions for someone else when those decisions influence the other person’s pay-offs and, secondly, how the decisions made for someone else compare to the choices made when completing the same task for oneself. Both the risk-as-feeling hypothesis (Loewenstein et al. 2001) and the Somatic Distortion Theory (Tunney & Ziegler, submitted) predict that people will be more risk seeking when making decisions for others than for self, because the emotional impact of the risky choices is reduced when the outcome affects a distant other.

## Methods

### Participants

Seventy-three participants [19 males (mean age 23.2 years ± 8.3) 54 females (mean age 20.1 years ± 1.0)] took part in return for a financial reward dependent on the choices made during the experiment. All participants were studying for a university degree at the University of Nottingham (N = 37) or the University of Lincoln (N = 36). The study was conducted with the approval of both Schools’ Ethics Committees.

### Experimental paradigm

Participants were presented with a variation of the financial decision-making task created by De Martino et al. (2006). Participants were tested individually. They were told in one part of the experiment that they would receive the payoffs of their decisions and in the other part that the payoffs would go to another anonymous participant. Participants were not informed of the second part until they had completed the first and the order of parts was counterbalanced. On each trial, participants were first told the amount of money at stake (e.g. £150). They were then presented with a sure option (framed as a gain or loss of a fraction of the money at stake, e.g. you keep (s/he keeps) £100 or you lose (s/he loses) £50), and a gamble option (with a pie chart representing the odds of winning vs. losing all the money at stake).

The choices between the sure and gamble options in experimental trials were between a 20, 40, 60 or 80 percent chance of keeping all the money with the sure offer matched to obtain the same percentage of the initial offer, so that the sure offer and gamble offer were equal in expected value. Eight filler trials in which the gamble option and eight in which the sure option had the greater expected value were interspersed to maintain participants’ motivation to make choices. A further eight control trials with probabilities to keep all the money of five and 95 percent respectively acted as catch control trials. In one block participants made decisions for self and in the other block they made decisions for another, with a practice session prior to the first block. Blocks were counterbalanced and participants were given no information about the second block of the experiment until the first was completed. Participants completed eight blocks of trials in the self and other conditions, each block consisting of four experimental, two filler and one catch trial. Each block was followed by interim feedback, indicating a running total of the amount of money won so far. To ensure that risk seeking would occur (Harinck et al. 2007), Participants played with larger virtual money offers of £150, £300, £450 and £600 that were converted into cash payments at the end of the experiment by dividing the grand total by 4000. At the end of the session participants were paid the money they had won in the self-condition, and money they had won in the other condition was placed into an envelope to be handed to the next but one participant. Participants received an envelope from the previous but one participant to make up their total pay. The first two participants therefore had to wait until the last and second to last participant had completed the experiment as their envelopes came from them.

## Results

**Figure 1:**
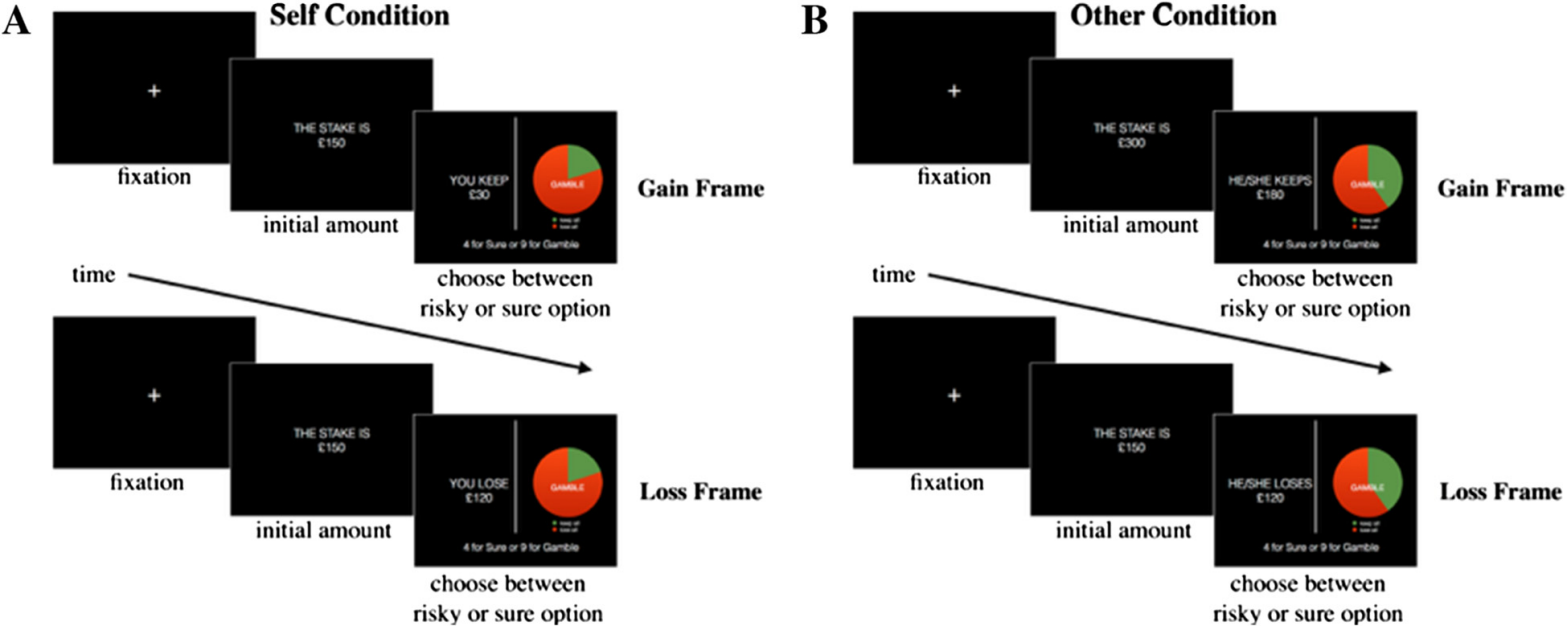
**The Decision-Making task.** Participants were first told the amount of money at stake (e.g. £150) and were told that they could never keep all of the initial stake. They were then presented with a sure option (framed as a gain or loss of a fraction of the money at stake, e.g. keep £100 or lose £50), and a gamble option (with a pie chart representing the odds of winning vs. losing all the money at stake). In the experimental trials the utilities of the sure option and gamble option were equal. Participants completed the task once for themselves (panel **A**) and once for an unknown other participant (panel **B**) with the wording of the framing reflecting this change in perspective.

Participants’ choices for self and other were converted to percentages of experimental trials in which they chose the gamble options in the loss or gain frames. Figure 1 shows the percentages of risky choices made for self and other in both loss and gain frames. The experimental trials had identical payoffs for sure and gamble options and therefore the effect of framing on decisions can be calculated from the percentage of trials in which participants chose the “gamble” option in the gain frame compared to the loss frame. In the gain frame the participants made more gambles for others than they did for themselves (other *M* = 39.93, *sd* = 16.18; self *M =* 36.48, *sd* = 16.19), and fewer gambles for others in the loss frame compared to gambles made for themselves (other *M* = 57.91, *sd* = 17.75; self *M =* 60.91, *sd* = 17.93). These data were entered into a repeated-measures ANOVA with the within-subjects factors of Target of the decision with 2 levels (self, other) and Frame of the choice with 2 levels (gain, loss). As a control the order of the conditions was entered as a between-subject factor.

There was a significant main effect of Frame (*F*_1, 71_ = 64.94, *MSE* = 506.22, *p* < .01, ${\eta}_p^2$ = .48), but no main effect of Target of the decision (*F*_1, 71_ < 1.0), or of the Order of conditions (*F*_1, 71_ < 1.0). There was, however, an interaction between Target and Frame (*F*_1, 71_ = 8.62, *MSE* = 87.27, *p* < .01, ${\eta}_p^2$ = .11). There was no main effect of Order or any interactions with Order of presentation (all *F*s *<* 1.0, except Target X Order: *F*_1, 71_ = 1.43, *MSE* = 105.22, *p* = .24, ${\eta}_p^2$ = .02). Paired sample t-tests reveal that the interaction is driven by a significant increase of gambling in the gain frame when making decisions for others compared to self (*t*_72_ *=* 2.01, *se* = 1.71, *p* = .04, Cohen’s d = .24), while the decrease in the gambles in the loss frame between self and other approaches significance (*t*_72_ *= −*1.96, *se* = 1.53, *p* = .05, Cohen’s d = .23).

**Figure 2:**
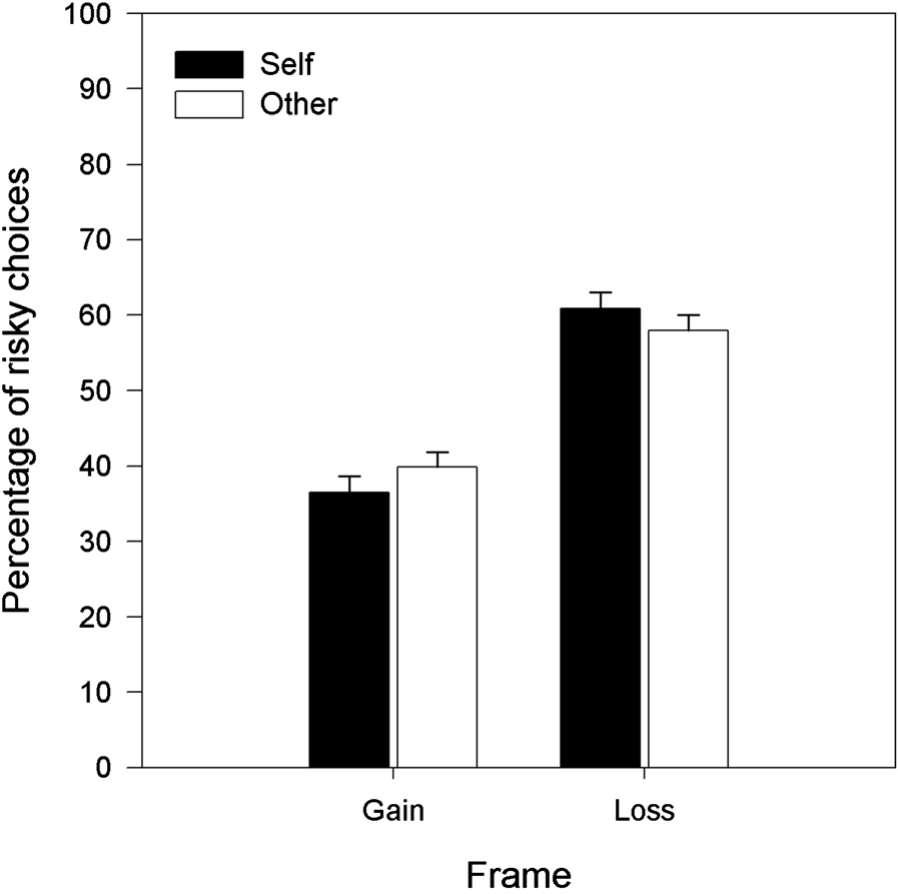
**Percent of risky choices by Target and by Frame.** Error bars are +1 standard error of the mean.

**Figure 3:**
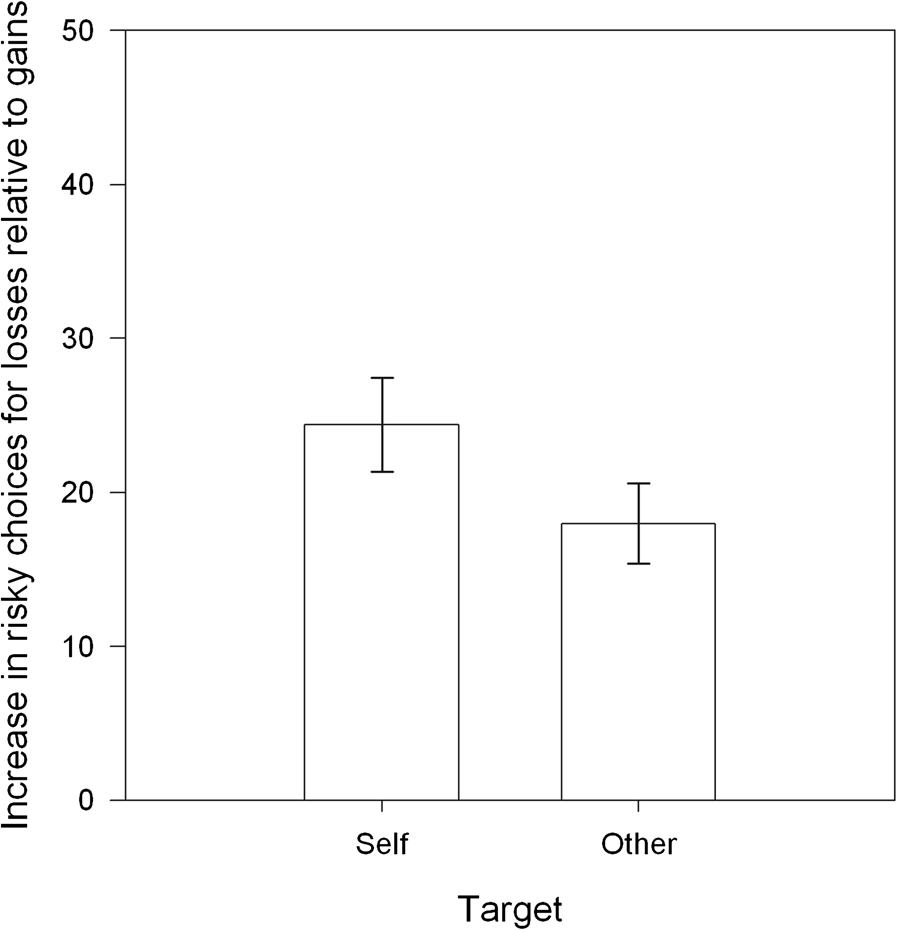
**Increase in risky choices for losses relative to gains by Target.** Error bars are +/− 1 standard error of the mean.

The susceptibility to the framing effect is best expressed as the difference of gambling choices made in the gain and loss frames; an increase in gambling choices in the loss frame signals risk seeking which is fundamental to the framing effect (Tversky and Kahneman 1981, 1986). The mean difference in risky choices made in the loss and gain frames for each target are shown in Figure 2. The increase in risky choices for losses relative to gains for self and other is shown in Figure 3. Although participants’ choices are subject to the framing effect in both conditions, there is a significant reduction of the effect when gambles are made for another person (*t*_72_, 2.95, *se* = 2.19, *p* < .01, Cohen’s d = .35), indicating that risk seeking, whilst present, is significantly reduced.

## Discussion

The experiment reported here investigated whether earning money for someone else affects risk seeking in a framed monetary gambling task. We presented participants with choices between sure and risky options in a series of gambles allowing them to win money; in one condition earning money for themselves and in the other earning money for another participant in the study. In contrast to Stone et al.’s (Stone et al. 2002) assertion that there are no differences in self and other decisions in monetary risk decisions we found that whilst framing occurred in both types of decision, there was a clear reduction in the framing effect when the decisions were not made for self.

How do our results speak to the questions of whether we are more risk seeking when making decisions on behalf of others? We found that participants were more risk seeking in making decisions for others compared to self but only in the gain frame. That is, when the options are framed as a gain then the predetermined outcome is preferred in self decisions compared to decisions made for others. In this sense, people are more risk seeking when making decisions for others. In the loss frame, however, they were less risk seeking when making decisions for others than they were for themselves. That is, risk seeking, whilst present, is significantly reduced when making decisions for someone else. Although, we have considered financial decisions in the laboratory a number of reports have documented similar effects for non-financial decisions involving risky behaviors or outcomes (Stone et al. 2013). Some of these (e.g. Wang 1996) that involve risk of death are similar to the original Disease Problem (Tversky and Kahneman 1981) and demonstrate the importance studying the more social aspects of decision-making such as this research to other fields such as medical decision making and aging research. Explanations for these social influences on decision making have included strategic game theoretical models (e.g.Trautmann and Vieider 2011; Tetlock 2002) or in terms emotional influences (Faro and Rottenstreich 2006, Tunney & Ziegler, submitted). There is some suggestion that the anonymity of the decision-maker relative to the recipient is also relevant with a degree of accountability or familiarity between the two people affecting the degree of bias (Pahlke et al. 2012; Hsee and Weber 1997; Ziegler and Tunney 2012). A number of other studies have found similar reductions in other cognitive biases when the decisions are made on behalf of other people (e.g. Fernandez-Duque and Wifall 2007; Garcia-Retamero and Galesic 2012; Jonas and Frey 2003; Nicolle et al. 2012; Ziegler and Tunney 2012).

There are other possible explanations for the observed reduction in framing effects for surrogate decision-making in the current study, which need to be addressed. One of the assumptions of Prospect Theory (Kahneman and Tversky 1979) is reference dependence, in that individuals identify a reference point representing their current state (Epley et al. 2004; Epley and Gilovich 2006). Gains and losses are therefore considered relative to this point. In surrogate decision-making, the current state of others is not known, so this potentially has to be approximated, possibly the reason for the decrease in framing effects when making decisions for others. Furthermore, other factors could also contribute to or be responsible for the reduction in framing effects for surrogate decisions. For example, Anderson’s (2003) Rational-emotional model, assumes that decision-making is influenced by factors that seek to reduce negative emotions, such as fear and anxiety. This is relevant to (De Martino et al. 2006) findings that show a role of the amygdala in decision-making and could explain what is meant by changes in ‘emotional involvement’. Furthermore the results of the current study could also be accounted for by a Social functionalist approach (Tetlock 2002) whereby the accountability and justification of the decision influences which decision is taken. If participants in the current study felt that they may possibly have to justify or be held accountable for their surrogate decisions, then this may also account for the reduction in framing effects. Further to this, studies such as (Wang 1996) have shown that the pattern of decision-making is influenced by social and moral factors, including the number of surrogates and the decision maker’s relationship to the surrogates. This relates to the social distance, as mentioned in the Background section, but this was discussed in light of delayed gratification and impulsivity.

## Conclusions

The data reported here complement a growing body of research that suggests that many traditional violations of rational choice theory can be reduced or eliminated when the decision environment is presented in format or context that can reduce decision biases. For example, a number of studies have shown that presenting gambles in frequency form can reduce base-rate neglect (e.g. Harries and Harvey 2000) and preference reversals (e.g. Tunney 2006). We believe that the bias that is reduced in the study reported here is one that is caused by a distortion in subjective utility estimation by an expectation of the emotional consequences of the decision. The subjective utility function in Prospect Theory itself gives an emotional account of the relative increase in risk seeking for losses relative to gains almost by definition of being subjective (Kahneman and Tversky 1979). It follows that by removing the personal relevance of the judgment one removes something of this emotional or somatic distortion and decisions become more normative and objective when they are made on behalf of other people.

The pattern of responses sits nicely within the Somatic Distortion framework. The emotional impact of the risk decisions is reduced when the outcome does not affect the decision maker directly. In the gain frame this means being less drawn to the certainty of the sure option and in the loss frame this causes a reduction in loss aversion and thus less risky gambling choices are made. We believe that this is the first report that the relative increase in risk seeking behaviour for loss frames is reduced when the decisions are made on behalf of other people, relative to when people make the same decisions for themselves. This is consistent with a general optimism about the subjective nature of cognitive biases, and a growing interest in the role that emotion plays in decision-making.
